# Challenges and opportunities of integrating noncommunicable disease prevention into maternal health services: A qualitative study

**DOI:** 10.1371/journal.pgph.0005683

**Published:** 2026-07-27

**Authors:** Doan T. T. Duong, Nga T. Q. Pham, Téa E. Collins, Duong M. Duc, Michelle L. Hermiston, Nguyen T. Tuan, Le T. K. Lam

**Affiliations:** 1 College of Health Sciences, Center for Innovations in Health Sciences, VinUniversity, Hanoi, Vietnam; 2 Vinmec International Hospital, Hanoi, Vietnam; 3 World Health Organization Vietnam, Hanoi, Viet Nam; 4 World Health Organization, Geneva, Switzerland; 5 Hanoi University of Public Health, Hanoi, Vietnam; Indiana University South Bend, UNITED STATES OF AMERICA

## Abstract

Pregnancy provides an opportunity to assess women’s health needs and engage them in appropriate healthcare services. This study explored how noncommunicable disease (NCD) prevention has been integrated into maternal health services in Vietnam and identified the barriers and facilitators influencing this process. We conducted in-depth interviews with 30 stakeholders, including policymakers, health managers, and providers in maternal healthcare services across different levels of care. Data were analyzed thematically using a conceptual framework. Recurring themes were identified and refined through discussions among investigators and technical advisors. Integrated NCD prevention and screening services were available before, during, and after pregnancy, particularly at higher-level public and private facilities, although mental health services remained limited. Limited primary healthcare capacity for maternal health and NCD prevention contributed to greater reliance on higher-level and private facilities. Most services packages relied on out-of-pocket payments rather than social health insurance coverage. Participants also identified gaps in service quality assurance, including counseling and continuity of care for women with NCDs, which may lead to inefficient service utilization and increased healthcare costs. NCD prevention services were integrated into maternal healthcare primarily at higher-level public and private facilities, whereas mental health care remained limited. Reliance on out-of-pocket payments, limited primary health care capacity, and gaps in continuity and quality of care may influence NCD prevention utilization and healthcare costs.

## Background

In 2023, more than 260,000 women died during pregnancy, childbirth, or the postpartum period worldwide. Most of these deaths were preventable through available cost-effective interventions [1]. Nearly 75% of maternal deaths are attributed to postnatal hemorrhage, postnatal infections, hypertension during pregnancy (pre-eclampsia and eclampsia), childbirth complications, and unsafe abortion [2]. Maternal deaths related to chronic conditions, including gestational diabetes and pre-existing or pregnancy-related hypertension, have also been increasing [2].

The global maternal health agenda has shifted from reducing maternal mortality to promoting women’s overall health and well-being. An important component of this shift is addressing maternal morbidity and the growing burden of NCDs among pregnant women. Pregnancy provides an open opportunity to access healthcare services, particularly those who may otherwise have limited healthcare utilization or may not seek treatment for chronic conditions. Maternal health services should therefore extend beyond emergency obstetric care to adopt a more comprehensive approach that encompasses preventive and early interventions, as well as integration with existing services [3]. Preventing hyperglycemia and hypertension in women has been identified by the World Health Organization (WHO) as a “best buy” intervention, underscoring the importance of prioritizing these measures in the prevention of NCDs [4].

In Vietnam, WHO estimates that NCDs are responsible for more than 80% of all deaths among women [5]. The prevalence of diabetes, hypertension, cervical and breast cancers, and mental health conditions among pregnant women and mothers has been increasing in recent year [6]. During pregnancy, these conditions are associated with adverse outcomes, including spontaneous abortion, stillbirth, congenital malformations, infant respiratory death syndrome, and birth injuries. Cardiovascular disease is the leading indirect cause of maternal mortality in Vietnam, accounting for 21% of maternal deaths [7]. These conditions may have long-term consequences for both maternal and neonatal health, with effects that can extend across generations [8].

Vietnam’s health system organizes into primary, provincial, and national levels, supports by vertical programs for maternal health and noncommunicable diseases (NCDs). Primary care facilities provide routine maternal care and uncomplicated deliveries, while women may also seek care at hospitals or private clinics. Pregnant women with complications, such as hypertension or gestational diabetes, are referred to higher-level facilities. Vietnam has achieved high utilization of maternal health services. In 2020–2021, 97% of pregnant women received at least one antenatal care visit, 88% had four or more visits, 96% delivered in health facilities, and 88% received postnatal care within two days [9].

Although maternal healthcare services were widely available in Vietnam, services for NCDs remain limited or underutilized [10]. Previous studies have primarily examined NCD services for the general population at the primary care level, with a focus on older adults [11–15]. One study explored the integration of maternal and mental health services at the primary healthcare level [16]. However, limited evidence is available on the provision of NCDs prevention within maternal health services, or about the enablers and facilitators that influence this process. This study aims to describe how noncommunicable disease (NCD) prevention has been integrated into maternal health services in Vietnam and identified the barriers and facilitators influencing this process.

## Materials and methods

### Ethics statement

The study was approved by the Institutional Ethical Review Board of Hanoi University of Public Health (Decision No 46/2023/YTCC-HD3 dated 07/02/2023). Informed consents were obtained from each participant before the interview.

### Study design and scope

This qualitative study used data from the WHO Review project in Viet Nam [17]. We explored the opportunities and challenges of integrating NCD prevention into maternal health services through in-depth interviews with key stakeholders. Prevention of NCDs included health promotion and screening for NCDs and their modifiable risk factors. Following the WHO approach to NCDs, we focused on five major conditions affecting women: cardiovascular diseases (CVDs), cervical/breast cancers, chronic respiratory diseases, diabetes, and mental health conditions [18]. Modifiable risk factors, including breastfeeding practices, nutrition, and physical activity, were considered across all stages of maternal healthcare, including pre-pregnancy, antenatal, and postnatal periods.

We adapted the framework for integrating NCD prevention into maternal health services for women, including pregnant and postnatal periods proposed by Firoz T in 2018 [3]. The framework proposed by Firoz et al. outlines essential healthcare interventions to address maternal morbidity, as described below; however, we excluded HBV vaccination and early detection/screening for HBV or HIV from our adaptation [3]:

- Prior to pregnancy care:
  - Counseling on the risk of specific contraceptive methods in women with specified NCD conditions; counseling for women with preexisting medical conditions, including preexisting diabetes and chronic hypertension.
  - NCD prevention should be promoted.
- Antenatal Care:
  - Promoting (counseling, mass media, etc.) NCD prevention includes breastfeeding (preventing breast cancer), physical activities, healthy nutrition, and avoiding harmful use of alcohol and smoking.
  - Early detection/screening for gestational hypertension, diabetes, mental health conditions
- Post-natal care:
  - Promoting NCD prevention, regular screening for breast cancer and cervical cancer, and breastfeeding
  - Screening for mental health conditions, breast cancer, and cervical cancer

**Fig 1:**
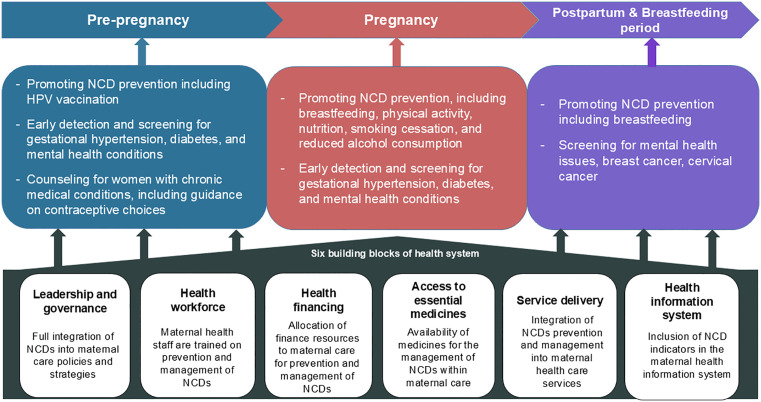
Conceptual framework for integrating NCD prevention into maternal health services.

We also incorporated the WHO six-building blocks framework for health system strengthening [19]. This framework includes (1) Leadership and governance; (2) Health workforce; (3) Healthcare financing; (4) Access to medical products, vaccines, and technologies; (5) Service delivery - promotion, early detection, and management of NCDs; and (6) Health information systems [19]. The conceptual framework is presented in Fig 1 which published in our previously report [17].

### Study setting and sampling

Vietnam’s Ministry of Health recognizes NCDs as a major public health challenge. National guidelines have been developed for the prevention and management of NCDs in the general population and among pregnant women. Updated technical guidelines for NCDs screening are available for: (i) gestational diabetes (Decision 1470/QĐ-BYT dated 29/05/2024); (2) pre-eclampsia (Decision no 1911/QĐ-BYT dated 19/04/2021), hypertension, pre-eclampsia, and eclampsia (Decision no 1154/QĐ-BYT dated 04/05/2024); (3) cervical and ovary cancer (Decision 2402/QĐ-BYT dated 10/6/2019), and (4) breast cancer (Decision 1639/QĐ-BYT dated 19/03/2021).

We consulted with National Professional Officers for noncommunicable diseases (NCDs) from the World Health Organization country offices and maternal health specialists from the Ministry of Health to select study sites and participants. Health care providers were purposively selected to ensure diversity in professional cadre (obstetricians, physicians, and midwives), service delivery context (antenatal care, childbirth, low- and high-risk pregnancy care), and level of care (national, provincial, and primary care).

Three major national obstetrics hospitals were included in the study. Hanoi Hospital of Obstetrics and Gynecology is a tertiary referral center in northern Vietnam that manages approximately 6,000 births annually. Tu Du Hospital is the largest tertiary obstetrics and gynecology hospital in southern Vietnam and manages approximately 60,000 births annually. Hung Vuong Hospital manages approximately 57,000 pregnancies annually and is recognized for integrating NCD services into maternal care.

At the provincial level, Thanh Hoa Province was selected to represent central Vietnam, including a provincial obstetrics and gynecology hospital. Within each study site, local Departments of Health assisted in identifying informants. Policymakers, government officials, and hospital managers involved in NCDs and/or maternal health services across different levels of care were also invited to participate.

### Data collection tool

We developed in-depth interview (IDI) guides based on the conceptual framework presented in Fig 1 above. Detail related to each health system building block—including health workforce, health information systems, medical products and technologies, financing, and leadership/governance) were explored as described below [19]:

- Service delivery: Types of NCD prevention services available across different levels of the health system.
- Leadership/governance: Oversight and implementation of NCD-related policies within the maternal healthcare system.
- Health workforce: Adequacy, distribution, competence, responsiveness, and productivity of healthcare staff.
- Information: Production, analysis, dissemination, and use of timely and reliable information on NCD risk factors, disease burden, and health system performance.
- Medical products, vaccines, and technologies: availability and access to essential medical products, vaccines, and technologies for NCD prevention and treatment
- Financing: Availability of adequate funding for health, health insurance coverage for NCD services within maternal healthcare, and incentives for providers.

A realist evaluation approach was applied to explore what works, for whom, how, under what circumstances, and in which contexts for each theme [20]. Interview guides were developed for each participant group and administered in Vietnamese. The guides were reviewed and agreed upon by technical advisors from the World Health Organization and the Ministry of Health before data collection. An example of the in-depth interview guide for service providers is provided in S1 Text. Ethical approval was obtained from the Institutional Review Board of Hanoi University of Public Health (No. 46/2023/YTCC-HD3).

### Data collection process

Participants were invited to join the study after being informed about the main interview topics. Written informed consent was obtained following verbal agreement to participate. Interviews were conducted at locations chosen by participants, such as offices or cafeterias, where confidentiality could be maintained. Each interview was conducted by two researchers with expertise in qualitative research, maternal healthcare, and health systems.

Data collection was conducted in four phases: (1) Hung Vuong Hospital; (2) Tu Du Hospital and lower-level facilities in Ho Chi Minh City; (3) Thanh Hoa Province; and (4) Hanoi. Approximately two weeks elapsed between each phase to allow for interview summarization, theme identification, refinement of the interview guides, and collection of additional information. After 30 interviews, no new themes emerged, and the research team agreed that data saturation had been reached (Table 1). Interviews were audio-recorded with participants’ permission; 27 of the 30 interviews were recorded. Detailed notes were taken for the three interviews that were not recorded and were cross-checked by both interviewers. Data were collected between 20 March and 12 April 2023. Each interview lasted approximately 45–90 minutes.

**Table 1:** Key informants in the qualitative study.

Key informants	Level	Representative	Number of IDIs
Medical staff working in maternal and child health care services	National-level hospitals (Tertiary Care)	Antenatal care and/or birth providers at the Hanoi Hospital of Obstetrics and Gynecology, Tu Du Hospital, and Hung Vuong Hospital (one in each hospital who oversaw antenatal care for healthy pregnancy and for high-risk pregnancy)	6
	Provincial level hospital (Secondary Care)	Antenatal care and/or birth providers including obstetricians and midwives in Provincial Obstetrics and Gynecology Hospital Thanh Hoa (one in each hospital who oversaw normal pregnancy care and one in high-risk pregnancy care)	2
	Primary care facilities	Antenatal care and/or birth providers in district hospitals	3
		Midwives in the commune health stations	3
Policymakers and managers	National level	Maternal and Child Health Department	2
		Preventive Medicine Department	1
	Provincial level (Ha Noi, Thanh Hoa, and HCM City)	Provincial health department	3
		NCD Department in Center for Diseases Control and Prevention	3
		Maternal and Child health Department in Center for Diseases Control and Prevention	3
	Hospital managers	Managers of Hanoi Hospital of Obstetrics and Gynecology, Tu Du hospital, Hung Vuong hospital, Thanh Hoa Provincial Obstetrics and Gynecology hospital	4
**Total**			**30**

### Data analysis

All interview recordings were transcribed verbatim. Two researchers independently reviewed the transcripts to ensure accuracy and completeness. Field notes and transcripts were coded and analyzed using NVivo (Version 11). The coding process was guided by the study data, research questions, and the thematic framework in Fig 1. Thematic analysis was used as it allowed for the extraction and interpretation of fundamental patterns, leading to the development of rich themes [21]. Thematic analysis was subject to the S2 Text researchers’ judgement; to mitigate this risk of bias. Quotes were first categorized by theme, and recurring ideas were grouped into subthemes. Relationships between subthemes were then examined to develop broader themes relevant to the research questions. Additional codes and themes are provided in).

### Researchers’ reflexivity

The research team comprised public health professionals with formal training in qualitative research and experience in maternal health and noncommunicable diseases-related studies. The primary researcher, a female public health researcher with expertise in maternal healthcare and health system research, led the study design, data analysis, and interpretation. Senior qualitative researchers (one male and three female) provided methodological oversight and guidance.

The researchers’ prior experience in maternal healthcare and health systems research may have influenced their perspectives and sensitivity to the study topics. Their professional backgrounds may also have affected data collection. On one hand, this experience helped establish trust and encouraged participants to share their views openly. On the other hand, participants may have assumed shared understanding and therefore provided less detailed explanations. To minimize potential bias, data collection was conducted in multiple phases with regular reflexive discussions among the research team. Interviewers used open-ended and non-leading questions throughout the interviews.

During the development of the interview guides and data analysis, researchers from the World Health Organization provided additional methodological input to strengthen analytical rigor. To enhance trustworthiness, findings were shared with stakeholders and presented at a hybrid consultative meeting organized by the Ministry of Health, where written feedback was obtained from participants.

## Results

### Availability of NCD prevention packages within maternal services in Vietnam at higher levels of care and in the private sector in Vietnam, except mental health care

A wide range of service packages integrating NCD prevention into maternal healthcare were available in major cities in Vietnam. These packages included health promotion and screening across the premarital, antenatal, and postnatal periods. Before pregnancy, all three national hospitals included in the study had developed premarital health promotion and screening packages covering hypertension, diabetes, breast and cervical cancers, and sexually transmitted diseases.

During pregnancy, services primarily focused on hypertension, preeclampsia, and diabetes. Medical staff performed blood pressure measurements and urine tests at every antenatal contact across all hospital levels in accordance with national guidelines. At provincial and national level, diabetes screening included urine testing at each antenatal visit, blood tests twice during pregnancy (once in the first trimester and once before delivery), and a glucose tolerance test at 24–28 weeks of gestation. National hospitals also offered preeclampsia screening packages for all pregnant women beginning in the first trimester:

“Hypertension is a condition that should be managed before pregnancy and throughout the antenatal, perinatal, and postnatal periods. All women who visit the hospital for antenatal care or giving birth are eligible for blood pressure measurement. To diagnose preeclampsia, all women are assessed for proteinuria” (IDI_NOH03_Manager01).

In the postnatal period, services were mainly about breastfeeding counseling and screening for breast and cervical cancers. Information on these service packages was available through hospital websites, YouTube, Facebook, leaflets, and billboards.

“There are different channels for promoting breastfeeding here, such as postnatal counseling, follow-up visits 42 days after birth, antenatal check-ups, and nutrition days. Occasionally, there are media broadcasts on breastfeeding.” (IDI_District02_Provider01)

Cervical cancer screening was provided as part of gynecological examinations at hospitals, whereas breast cancer screening was offered through separate service packages:

“Cervical cancer screening is offered to all women attending health facilities for gynecological examination at all levels. We used Pap smears or VIA for screening. For pregnant women, we do not routinely perform this screening unless there are suspicious signs.” (IDI_CDC02_Manager02)

However, screening and referral services for women with perinatal mental health conditions were lacking. No formal guidelines were available for counseling and screening of maternal mental health conditions. Providers often relied on personal experience to identify symptoms of mental health problems, despite lacking appropriate training:

“When patients ask questions about abnormalities of the unborn baby or appear excessively worried about something, I feel that they may have mental health issues. That is based on experience.” (IDI_POH01_Provider_2)

Maternal healthcare providers also reported limited capacity to manage potential mental health conditions.

“We have no time to answer questions, or the answers we provide are not adequate. We cannot fully meet the needs of pregnant women if they have concerns about mental health. Even if we identify mental health problems, we can do nothing.” (IDI_POH01_Provider_1)

“This [mental health] is a major gap. We are not doing anything about it, and no one asks or pays attention because there are too many patients. Doctors do not have enough time to ask women about their experiences or how they are feeling.” (IDI_NOH01_Provider02)

### Limited availability and utilization at the primary health care level

The primary health care level included district health facilities (district hospitals, district health centers) and community health stations. Service packages before, during, and after delivery were available at district health facilities. However, these services were less comprehensive and had limited opportunities for promotion. Community health stations provided primarily health promotion services and referred women to higher level facilities for services.

#### Limited human resource capacity for NCD prevention at lower levels of care.

Healthcare workers with the capacity to diagnose and manage noncommunicable diseases (NCDs) were mainly concentrated in major cities and tertiary-level facilities. At the provincial level, human resource capacity remained limited because of insufficient training and lack of guidance from experienced specialists. Health workers at district and commune health facilities also had limited understanding of NCDs. Qualitative interviews highlighted these challenges:

“We have a plan to implement the NCDs program at the commune level. The greatest difficulty is human resources. Staff need in-depth knowledge of NCDs. They do not clearly understand what NCDs are; it is easier to mention specific diseases or risk factors such as hypertension and diabetes. Health staff need training in NCDs.” (IDI_CDC02_Manager03)

Interviews with healthcare providers at the commune health stations revealed that few pregnant women attended these facilities for antenatal care, particularly in urban areas where public and private obstetric clinics and hospitals were readily available. Women often preferred higher-level facilities because they perceived the care to be more comprehensive and safer.

“No one comes here for antenatal contact anymore. It used to happen, but since 2014, when the commune health station stopped supporting childbirth, women no longer came for antenatal care or counseling.” (IDI_Commune02_Provider1)

“Currently, we want to strengthen noncommunicable disease management at commune health stations. However, the management of pregnant and postnatal women at this level is currently in crisis. Frankly speaking, the situation is extremely poor. Only 10% to 20% of commune health stations have pregnant women attending antenatal care.” (IDI_CDC02_Manager03)

Because maternal healthcare staff at the provincial level had limited expertise in NCD management, women with gestational diabetes, hypertension or other NCDs were often referred to the provincial or national hospital for re-testing and counselling. After the initial diagnosis, routine follow-up management of NCDs were generally not integrated into maternal healthcare services.

#### Limitations in funding and local government commitment to strengthen NCD prevention, including among women during the perinatal period.

During the 2021–2025 period, state funding for both the NCD and reproductive health programs was limited. Implementation of these programs largely depended on local government support. In large cities such as Hanoi City and Ho Chi Minh City, local budgets were available; however, funding priorities were more focused on NCDs than on reproductive or maternal healthcare services.

“Budget is available. However, Hanoi has already achieved sustainable development targets. The indicators of maternal mortality, under-five mortality, and under-one-year mortality have all reached the targets. Hanoi’s indicators are comparable to those of high income countries. MOH recommends at least four antenatal contacts but in Hanoi women often attend around 10 visits.” (IDI_CDC03_Manager01)

Participants also noted that not all local governments had dedicated health budgets comparable to those of large cities such as Hanoi and Ho Chi Minh City.

“For localities that depend on the state budget, the Ministry of Health must support them through national target programs aimed at reducing poverty and inequities, particularly in mountainous ethnic minority provinces.” (IDI_MOH02)

Limited budgets and delays in funding allocation were reported as barriers to implementing community-based cervical screening programs:

“Currently, the programs have no funding [to invite people to attend screening]. If we provide something for them, such as supplements or even leaflets, they participate; if not, they do not. This makes implementation difficult.” (IDI_Commune01_Provider01)

“In 2022, funding was allocated very late, so we could not implement prevention activities at the commune health station. We were only able to organize some training activities to keep the program on schedule.” (IDI_CDC01_Manager01)

### Hospital autonomy and the expansion of private clinics increased the availability and accessibility of antenatal care services

The expansion of private clinics and hospitals, together with the fiscal autonomy of public hospitals, has increased the availability and accessibility of maternal services. Health facilities have introduced new service packages to strengthen their competitiveness and attract clients. Competition among facilities was also perceived to improve service quality, particularly counseling services:

“In previous years, hospitals were quite crowded; however, more private clinics have now opened, and the number of patients has decreased. Many women prefer to visit private clinics. With fewer patients, doctors have more time to provide counseling.” (IDI_POH01_Provider 02).

Hospital autonomy also enabled managers at the central level to actively mobilize and allocate funds for hospital marketing and health promotion activities:

“Hospitals allocate funding for health promotion activities. In addition, we mobilize support from companies for promotion and screening service packages. For example, we may use their products for women with gestational diabetes mellitus to support diet management and blood glucose control.” (IDI_NOH01_Manager02)

As a result, clients often preferred private over public antenatal care services. Reasons included shorter waiting times, fewer administrative procedures, better provider attitudes, and more advanced equipment, particularly ultrasound technologies. The mechanism allowing obstetricians to work in both public hospitals and private clinics, together with the option for women to choose specific healthcare providers to support childbirth at public hospitals, facilitated continuous follow-up throughout pregnancy and childbirth.

“Women go to private clinics [for antenatal care] because the obstetricians working obstetrics hospital often have their own clinics. Women can choose an obstetrician to support them during childbirth at the hospital. When women experience signs of labor, they can contact the obstetrician, who advises them on when and how to go through hospital’s procedure. This is convenient and timesaving. Public hospitals are often very crowded.” (IDI_Commune01_Provider01)

Women often choose district hospitals or private clinics with skilled and well known obstetricians. Some women even choose provincial or higher-level facilities [for antenatal care or childbirth].” (IDI_CDC02_Manager03)

### Reliance on out-of-pocket payment and limited insurance coverage

Despite policies supporting screening, health insurance did not cover most preventive services including NCD, such as nutrition examinations and counseling, or screening for early cancer detection.

Before pregnancy, the service was referred to as “premarital medical counseling and examination,” it was perceived as targeting young people before marriage rather than as pre-pregnancy care. More importantly, it was not covered by health insurance and often received limited attention until women experienced fertility or conception problems. As the results, uptake of these services remained low, resulting in missed opportunities for preconception screening among many women.

“All women should have a preconception examination. For example, women undergoing in vitro fertilization need to be examined before becoming pregnant. Once they become pregnant, they do not need to repeat the examination. However, many women miss preconception examinations…It would be better if they were screened from the beginning, before pregnancy.” (IDI_NOH01_Provider02)

Although antenatal care visits were covered by health insurance, many screening tests, such as those for preeclampsia and gestational diabetes, were not. As a result, NCD counseling and screening services were primarily available to women who could afford out-of-pocket payments.

“Public hospitals serve many different types of patients, including those with good economic conditions and those with financial difficulties. Counseling on this service packages must consider patients’ financial status. It depends greatly on whether patients can afford to pay for them.” (IDI_NOH03_Provider01)

“For screening and counseling activities, the biggest challenge is that they are not covered by health insurance.” (IDI_MOH01)

As a result, pregnant women often underwent opportunistic NCD screening only during antenatal care visits.

### Challenges in ensuring access to appropriate counseling for women

Obstetricians reported that effective counseling can help promote the screening packages, benefiting both patients and hospitals.

“I spend time on counseling because if the patient agrees to use the screening packages, it increases the benefit to the hospital. In addition, it also helps to increase the number of patients for my private practice” (IDI_NOH01_Provider01).

However, concerns were raised about the quality of counseling provided during pregnancy and the postnatal period, which varied across healthcare providers. Participants noted the absence of mechanisms to ensure the quality and consistency of counseling services.

“The number of patients is too large, so consultation and communication are limited. Medical staff do not have enough time for this.” (IDI_NOH03_Manager01)

“When there are many patients, the consultation process is not thorough. If there are fewer patients, providers have more time for counseling. With many patients, they often provide counseling to a group before conducting the check-up.” (IDI_District02_Provider01)

Maternal healthcare providers mainly counseled women on screening and managing pregnancy-related complications, including hypertension, preeclampsia, and diabetes. Women diagnosed with hypertension or diabetes received additional counseling on nutrition and dietary management, although these services were mainly available at national hospitals:

“I advise pregnant women whose blood pressure is higher than normal. Even if they do not ask questions, I still provide advice. If they ask questions, I give more detail counseling.” (IDI_POH01_Provider02)

“For pregnant women with diabetes, there is a counseling and glucose testing room. If a patient has a positive glucose test, she receives counseling on dietary management.” (IDI_NOH01_Manager01).

Nevertheless, limited consultation time remained a major barrier.

“There is a protocol for antenatal check-ups that we must follow. However, as this hospital is the highest level of care, it is difficult to provide thorough counseling because the time available for each patient is limited.” (IDI_NOH02_Manager01)

Counseling NCD risk factors, including nutrition and physical activity across all stages of maternal care (pre-pregnancy, antenatal, and postnatal), was limited:

“I must admit that the counseling for women with uncomplicated pregnancies is extremely limited. We only briefly mentioned nutrition or physical activities. Only women with complications or conditions such as gestational diabetes receive more detailed counseling from both obstetric staff and nutrition specialists in the hospital.” (IDI_NOH03_Provider01)

According to the National Guidelines for the Provision of Reproductive Care Services, healthcare workers providing contraceptive methods should counsel women carefully and screen for diseases and conditions, including NCDs, across all levels of maternal and child healthcare services. In practice, however, contraceptive services were mainly provided to married postpartum women and rarely considered the presence of NCDs. Participants also noted the lack of communication materials on contraceptive methods for women with chronic conditions:

“Communication materials are available on topics such as nutrition, diabetes, and contraceptive methods, but materials on contraceptive methods for women with chronic diseases are not available.” (IDI_CDC03_Manager01)

## Discussion

In Vietnam, a wide range of service packages for NCD prevention and management before, during, and after pregnancy has been provided at national-level obstetric hospitals. These factors create a favorable environment for integrating NCD prevention into maternal health services. However, important gaps remain in guidelines and care pathways for women living with NCDs and mental health conditions, despite the rising prevalence of these conditions among women [3]. Guidelines and care pathways for women with mental health conditions are urgently needed.

While primary healthcare facilities were commonly used for general illnesses, the capacity of Vietnam’s primary care system to address NCD-related health needs remained inadequate, and NCD services were underutilized [12,22,23]. Our findings showed limited availability and utilization of both NCD and maternal healthcare services at the primary healthcare level, consistent with previous studies conducted in urban and rural areas of Vietnam [13,14,24]. Reported challenges included shortages of healthcare workers, limited numbers of staff trained in NCD management, and insufficient funding and commitment from local governments for NCD services within primary healthcare [13,14,24]. In addition, integrated NCD management was not routinely included in training programs, and adherence to clinical guidelines remained low [25].

Healthcare providers reported that women preferred higher-level and private maternal healthcare services, consistent with previous studies [26,27]. The increasing use of higher-level and private maternal healthcare services may have important implications for health systems, equity, quality of care, and healthcare costs. In Vietnam, overcrowding at tertiary hospitals has been linked to patients bypassing lower-level facilities, resulting in prolonged waiting times and increased pressure on specialist services, for example [28]. Preferences have increased, particularly among wealthier groups [29]. In mountainous and disadvantaged regions, many women traveled farther and paid out of pocket for private or higher-level maternal healthcare services; for example, nearly 60% sought uninsured antenatal care at private clinics [27,30].

Financial autonomy has created opportunities for both public and private sectors to expand and improve the availability and continuity of maternal healthcare services. However, concerns remain regarding service regulation and quality [31]. Hospital autonomy may also incentivize revenue-generating practices, including the provision of “patient-requested” services [32]. In addition, private services and higher-level facilities often require substantial out-of-pocket payments, particularly for screening and preventive services not covered by health insurance. Although the contribution of hospital autonomy and the private sector to expanding the integration of NCDs into maternal healthcare services remains unclear, the associated financial burden and inequities are evident. Despite health insurance covered reaching 91% of population in 2020 [33]; women still had to pay a substantial proportion of healthcare costs out of pocket [34]. The service packages are relied on out-of-pocket payments and the limitation of the primary health care system in providing services of both maternal health and NCD prevention are accelerating the utilization of services in higher levels of care or the private sector. These patterns contribute to discontinuity of care, loss of follow-up, and exacerbate health inequities, risk, and vulnerability within society.

Counseling remains one of the most neglected components of maternal healthcare, particularly regarding nutrition and physical activity during pregnancy, as reported in previous studies [7]. Monitoring data on counseling, communication, screening, and targeted NCD interventions are also less consistently reported than data on disease management and treatment [35]. In addition, communication on NCD risk factors remains insufficient, and health workers are not widely recognized as trusted sources of health information [11].

Several factors may undermine the quality of counseling services, including power asymmetries between healthcare providers and patients, financial incentives associated with counseling and screening services, and the concurrent practice of healthcare providers in both public hospitals and private clinics. Maternal healthcare providers often deliver information in a didactic, one-way manner and rely heavily on written materials rather than interpersonal communication. Consequently, women must take a more active role in seeking information relevant to their needs, while facing additional barriers such as language differences, gender dynamics with providers, limited consultation time, and reluctance to ask questions [36]. Previous research on gestational diabetes mellitus described the condition as “biomedically present but socially absent,” as family members often perceived it as insignificant or non-existent [37]. This created additional challenges for women’s self-care and placed them in difficult social positions. Addressing these barriers requires broader organizational transformation beyond workflow or policy changes alone [38]. These findings highlight the need for patient-centered counseling approaches, stronger provider communication training, improved quality assurance mechanisms, and better regulation of financial incentives and dual public–private practice within maternal healthcare services.

### Limitations

There are potential biases in that participants may have provided socially desirable responses or been reluctant to report malpractice involving themselves or their colleagues. To minimize this bias, we selected a diverse range of stakeholders to ensure comprehensive perspectives and adequate information for the study. Our study did not include women or family members because the scope was limited to understanding systemic factors. However, their perspectives may have provided deeper insights into patient–provider interactions. Further research is needed to identify optimal strategies to reduce inequities and improve the quality of NCD prevention within maternal healthcare services.

## Conclusions

A wide range of NCD prevention services before, during, and after pregnancy were available in Vietnam, although mental health services remained limited. Hospital autonomy, private sector expansion, and weaknesses in primary healthcare appeared to increase the availability of fee-for-service packages, while gaps in insurance coverage for preventive services may worsen inequities and financial burdens. Mechanisms to ensure quality counseling and continuity of care for women with NCDs were also lacking. Our findings highlight the need for standardized national guidelines and referral pathways, strengthened primary healthcare capacity, ensured counseling quality, and expanded insurance coverage to improve the continuity and quality of integrated maternal and NCD care, including perinatal mental health services.

## Supporting information

S1 Text Interview Guide. (DOCX)

S2 Text Additional codes. (DOCX)

## Abbreviations

HPV: human papillomavirus
HIV: human immunodeficiency virus
MOH: Ministry of Health
NCD: non communicable diseases
WHO: World Health Organization.
