# Supplementary material for: Challenges and opportunities of integrating noncommunicable disease prevention into maternal health services: A qualitative study
Source: PLOS Glob Public Health. 2026 Jul 27;6(7):e0005683. doi: 10.1371/journal.pgph.0005683 (PMC13405284; doi:10.1371/journal.pgph.0005683)
Supplement: S1 Text — (DOCX) [file pgph.0005683.s001.docx]

**In-depth Interview Guide**

**Study on the Integration of Prevention and Management of Noncommunicable Diseases into Maternal Health Services in Vietnam**

**Interview participants**

Service providers

**Objectives**

1. To describe the status of integrating prevention and management of noncommunicable diseases (NCDs) into maternal health services in Vietnam.
2. To identify facilitators and barriers to integrating prevention and management of NCDs into maternal health services in Vietnam.
3. To provide recommendations to improve the integration of NCD management into maternal health service delivery in Vietnam.

**Interview Content**

## 1. Before pregnancy

**Counseling**

What counseling activities related to NCDs are currently provided for women before pregnancy? At which levels can counseling be provided (commune health stations, district health centers/hospitals, provincial hospitals)? Who can provide counseling? What is the current capacity of health workers to provide counseling?

In some countries, integrated counseling for women before pregnancy includes the following topics:

| **Counseling content** | **Feasibility** |
| --- | --- |
| Risks of contraceptive methods for women with specific NCDs |  |
| Women with underlying conditions such as diabetes, hypertension, sickle cell disease, cardiomyopathy |  |
| Counseling diet and physical activity to improve health and prevent NCDs |  |

Please describe the feasibility of implementing these activities at your facility. What challenges may arise? What additional infrastructure or equipment would be needed?

**Health communication**

In some countries, communication activities on NCDs are provided for women before pregnancy. Please describe the feasibility of implementing these activities at your facility. What challenges may arise? What additional infrastructure or equipment would be needed?

**Screening**

Which NCDs can currently be integrated into screening before pregnancy? When should screening occur? At which levels can screening be conducted? Who can conduct screening? What is the current screening capacity of health workers? What additional infrastructure or equipment is needed?

Note: Clarify what is currently being done, the facilitators and barriers, and what could be implemented if challenges were addressed.

**Management and treatment**

Which NCDs are currently managed and treated before pregnancy? If a woman is suspected of having hypertension, diabetes, or depression, what is the management process (confirmation of diagnosis, treatment, follow-up, referral, etc.)?

At which levels can management and treatment be provided? Who can provide these services? What is the current capacity of health workers? What challenges are encountered? What additional training, infrastructure, or equipment is needed?

## 2. During pregnancy

**Counseling**

What counseling activities related to NCDs are currently provided for pregnant women? At which levels can counseling be provided? Who can provide counseling? What is the current counseling capacity of health workers?

In some countries, integrated counseling and communication during pregnancy include:

| **Counseling content** | **Feasibility** |
| --- | --- |
| Breastfeeding |  |
| Physical activity |  |
| Nutrition |  |
| Alcohol and tobacco use |  |
| Communication activities |  |
| Others |  |

Please describe the feasibility of implementing these activities at your facility. What additional infrastructure or equipment would be needed?

**Screening**

Which NCDs are currently screened during pregnancy?

Ask specifically about screening for hypertension, diabetes, and mental health conditions. When is screening conducted? At which levels? Who conducts screening? What is the current screening capacity of health workers? What are the facilitators and barriers? What additional infrastructure or equipment is needed?

**Management and treatment**

How are NCDs currently managed and treated during pregnancy? If a pregnant woman is suspected of hypertension, diabetes, or depression, what is the management process (diagnosis, treatment, follow-up, referral, etc.)?

At which levels can management and treatment be provided? Who can provide these services? What is the current capacity of health workers? What facilitators and barriers exist? What additional training, infrastructure, or equipment is needed?

## 3. After childbirth and during breastfeeding

**Counseling**

What counseling activities related to NCDs are currently provided for postpartum women? At which levels can counseling be provided? Who can provide counseling? What is the current counseling capacity of health workers?

In some countries, integrated counseling and communication for postpartum women include breastfeeding promotion and NCD prevention. Please describe the feasibility of implementing these activities at your facility. What facilitators and barriers exist? What additional training, infrastructure, or equipment is needed?

**Screening**

Which NCDs are currently screened for postpartum women?

Ask specifically about screening for mental health conditions, breast cancer, and cervical cancer. When is screening conducted? At which levels? Who conducts screening? What is the current screening capacity of health workers? What facilitators and barriers exist? What additional training, infrastructure, or equipment is needed?

**Management and treatment**

How are management and treatment currently implemented, including detection, referral, and follow-up across levels of care?

At which levels can management and treatment be provided? Who can provide these services? What is the current capacity of health workers? What facilitators and barriers exist? What additional training, infrastructure, or equipment is needed?

## 4. Facilitators and barriers to integrating NCD care into maternal health services in Vietnam

Besides the facilitators and barriers mentioned above, are there any additional factors influencing the integration of NCD care into maternal and child health services in Vietnam?

**Policy and governance**

Which current policies address the integration of NCD prevention and control into maternal health care? Which components are still lacking? How are these policies being implemented?

**Health information systems**

What NCD indicators among women are currently collected and reported? At which levels and at what reporting intervals? What is the reporting process? Which indicators are still needed to improve planning and policy development?

Examples:

- Detection and treatment follow-up rates for hypertension and diabetes among women
- Indicators for pregnant and breastfeeding women
- Mental health indicators among pregnant, postpartum, and breastfeeding women

**Human resources**

What are your general views on the number and capacity of health workers for integrating NCD care into maternal health services, including counseling, screening, and referral? What additional training is needed?

**Infrastructure and equipment**

To what extent do current infrastructure and equipment support counseling, communication, and screening activities at provincial, district, and commune levels?

**Financing**

How is funding currently allocated for integrated services? Are there dedicated budgets for programs such as nutrition, breast cancer prevention and control, or cervical cancer prevention programs?

## 5. Recommendations

What recommendations do you have to strengthen the integration of NCD management into maternal health service delivery in Vietnam?
