# Supplementary material for: Challenges and opportunities of integrating noncommunicable disease prevention into maternal health services: A qualitative study
Source: PLOS Glob Public Health. 2026 Jul 27;6(7):e0005683. doi: 10.1371/journal.pgph.0005683 (PMC13405284; doi:10.1371/journal.pgph.0005683)
Supplement: S2 Text — (DOCX) [file pgph.0005683.s002.docx]

# S2. Additional quotes to support the findings

## Service delivery

### Type of services and time

#### Pre-pregnancy care

All three national hospitals had developed their packages and provided premarital counseling and examination which included high blood pressure, diabetes, breast and cervical cancer, and mother-to-child transmitted diseases. The national gynecology hospitals could attract several people having premarital examinations. They also provided a premarital examination for free under the program of the Provincial Department of Health.

*There are quite a lot number of people who are seeking premarital examination. It was about 30 – 50 people per day. Besides, we also participated in providing premarital examinations for free under the Provincial Department of Health in Can Gio district. It is to support young married couples who are poor, or near poor. The package has everything including medical tests to ensure their ability to have a child.* (PVS_NOH01_Manager01)

Premarital examinations were also provided at provincial and district hospitals but in less comprehensive packages and less promotion. Commune health stations are providing premarital counseling and communication, and refer people to higher levels for examination. Premarital communication could provide at the high schools as extracurricular activities depending on the plan of the commune but there were quite a few people who visit commune health centers just for receiving premarital counseling.

To control NCDs, the premarital examination was considered important and needed to be taken as early as possible to ensure the well-being of women, not until before getting married or preparing for pregnancy by healthcare providers and managers.

*All women should have had a pre-pregnant examination. For example, if women have IVF, they will be offered a pre-pregnant examination before becoming pregnant. Once they get pregnant, they don't need to do it again. But women are missing pre-pregnant examinations so when they get pregnant it's a big problem. So it would be better if they were screened from the beginning, before having a pregnancy.* (PVS_NOH01_Provider02)

#### Antenatal care

##### **High blood pressure**

Blood pressure measurement and urine testing were conducted in every antenatal visit to screen for increased blood pressure and pre-eclampsia in all studied hospitals. Blood pressure measurements often were conducted by midwives and urine was tested by technicians. The result of the blood pressure measurement was written down in the pregnant visit record and the urine testing record was attached.

“Hypertension is a symptom that must be managed before and during pregnancy, at birth, and after birth. All women who come to the hospital for antenatal visits and giving birth are eligible for blood pressure measurement. To diagnose preeclampsia, all of them are tested for proteinuria” (PVS_NOH03_Manager01)

The Ministry of Health recommends pregnant women have at least four pregnancy visits, one in the first trimester, two in the second trimester, and one in the last trimester. However, in those hospitals at the national or provincial level, mothers were recommended to have at least 10 – 14 times.

All women were counseled for pre-eclampsia screening packages since they were in the first trimester. The counseling was conducted either by a midwife or mainly by an obstetrician. The content and price for pre-eclampsia screening packages were varying among hospitals ($12 - $55). Women would receive the results of the screening in their next antenatal visit if they were at low risk of pre-eclampsia. In case of high risk, they would receive counseling and treatment right at this antenatal visit.

Pregnant women who had high blood pressure would receive counseling and treatment options.

“I advise pregnant women who have a blood pressure higher than normal. If they do not ask, I still give advise. If they know how to ask, I will give advice more carefully and in detail.” (PVS_POH01_Provider02)

Regarding treatment, the hospital treats hypertension and pre-eclampsia according to the hospital's protocol and guidelines from the Ministry of Health. Women who had pre-eclampsia, for example, would be asked to sign a consent form for prophylactic aspirin treatment in the Hung Vuong hospitals. In general, most hypertension cases would receive treatment and follow-up at national obstetrics hospitals. Obterician described blood pressure medications and magnesium sulfate (in case of preeclampsia) and asked them to visit the national obstetrics hospitals every week for testing. Very few cases were referred to a heart and vascular hospital for treatment (0.9% - 13%).

“The hospital treats hypertension and pre-eclampsia according to the hospital's protocol. Severe cases of hypertension are transferred to Hanoi Heart Hospital or Heart Institute in Bach Mai Hospital”. (PVS_NOH03_Manager01)

Provincial and district hospitals referred more hypertension cases to a heart and vascular hospital for treatment. Pregnant mothers who had hypertension before pregnancy or were unsure about the causes of hypertension or severe cases would be referred to a heart and vascular hospital for treatment.

“A: If pregnant women have high blood pressure due to pregnancy, we keep them in the hospital for monitoring, and if they have hypertension before pregnancy, we refer them to the cardiologist for monitoring.

Q: What if the woman has never been to a cardiologist?

A: Then we refer them to the cardiology department of the general hospital”

(PVS_POH01_Provider01)

There was no follow-up system between the obstetrics hospital and the heart and vascular hospital.

##### **Diabetes**

Diabetes was screened through urinalysis in each antenatal visit, blood testings twice (one in the first trimester and one before delivery), and a glucose tolerance test at 24-28 weeks of pregnancy. The glucose tolerance test sometime was refused by pregnant women in Thanh Hoa province because of time-consuming:

“The glucose tolerance test takes time, at least 2 – 3 hours. Women need to skip their breakfast and wait for a long time. In Thanh Hoa, most of them are workers, they just want to do a quick test.” (PVS_POH01_Manager01)

In national obstetrics hospitals, there was a nutrition department that provided counseling and a diet for pregnant women with abnormal glucose testing to follow at home. In case of no improvement, pregnant women would be hospitalized for management, mainly on diet.

“For pregnant women with diabetes, there is room for counseling, screening, and sugar testing. If the patient has a positive glucose test, they will be counseled on food restriction. The Department of Nutrition will advise the mother. If the patient's performance is not stable, then they will be admitted to the hospital. Patients who have positive glucose tests and are admitted to the hospital will be on a diet and monitored by the doctor. The hospital also prepares meal following the diet for those patients and help them to self-monitor their room. Doctors also monitor and advise and only use insulin when it's severe.” (PVS_NOH01_Manager01)

Obstetricians needed to refer pregnant women to endocrine hospitals for describing insulin treatment except if they had a certificate for doing so. There were very few obstetricians who had that certificate, mostly in Hung Vuong Hospital. The referral rate of Hung Vuong Hospital was only 3% compared to 20% of Tu Du Hospital.

“If a woman has diabetes before pregnancy or before 12 weeks of pregnancy, I will refer her to an endocrinology hospital so they can evaluate for complications of diabetes or evaluate for gestational diabetes, or do glucose tolerance therapy to guide her on a diet. Depending on each case and her risk factors, we re-evaluate whether she responds well to the diet or not. In case there are too many risk factors affecting the baby or caused by family history, we will send her again to endocrinology for insulin treatment.” (PVS_NOH02_Manager02)

Hanoi Obstetrics and Gynecology Hospital was similar to Tu Du Hospital. She referred those need insulin treatment to the National Endocrinology Hospital or the Department of Endocrinology at Bach Mai Hospital. Two obstetricians here were obtaining a certificate for insulin treatment. All pregnant women who were found positive for diabetes in Thanh Hoa Provincial Obstetric Hospital would be referred to endocrine hospitals. Currently, there was no follow-up system for those patients. They would be back to the obstetrics hospital for antenatal care or delivery or select another one.

##### **Mental health care**

Mental issues were not integrated into maternal health care services. There were no policies and guidelines on screening mental health for pregnant women. Maternal health care providers received no training in mental health screening, detection, and management, they detected mental health symptoms based on their experience:

“When the patient asked a lot of questions about abnormalities of the unborn baby, or they were too worried about something, I feel that they may have mental health issues. That is on experience.” (PVS_POH01_Provider_2)

In addition, pregnant women hesitated to share their feelings about their fear of healthcare providers having no time for counseling and being able to do nothing:

“It’s very rare. Almost no one asks about mental health issues. A lot of patients are waiting. Doctors have no time to answer, or their answers are not adequate. It cannot fulfill the need of pregnant women if they have concerns. Pregnant women, therefore, are not trusted and do not ask questions. Even if we found out about mental health problems, we can do nothing.” (PVS_POH01_Provider_1)

Maternal health care providers also shared the time constraint in asking and counseling for mental health issues:

“This [mental health] is a big gap, we also do nothing about it, no one asks or cares because there are too many patients. There was not enough time for the doctor to ask what the woman did and then how her psychophysiology was.” (PVS_NOH01_Provider02)

Besides time constrain, low perception of mental health issues and no clear treatment pathways made maternal healthcare providers ignore mental health issues

“Mental health issues are very delicate, when I realize this woman has a problem, I tell them or their family, they say doctor crap. The fact that mental health is quite ambiguous. There are a lot of mental health scales with different results. So when I hold a prescription for one patient, the other person says it's okay to use or not to use. Therefore, the patient does not know what to do.” (PVS_NOH01_Provider02)

Pregnant women, if they shared their mental health symptoms with maternal health care providers, would receive advice on relaxing and nutrition. The treatment mainly focused on the relief of physical symptoms related to pregnancy status. If mental health symptoms were severe and obvious, pregnant mothers would be advised to a higher-level obstetrics hospital, not mental health hospital at a higher level because of their pregnancy status.

“If we see that pregnant women cannot rest their minds, we will have to refer them to higher-level hospitals. We will not dare to manage them at these district-level facilities. We often refer them to the obstetric department of the provincial hospital. We know [psychiatric hospitals] but dare not recommend them to go straight to those facilities. Because they are pregnant and we don’t know much about the matter, so we don’t dare to introduce them [to psychiatric hospitals] and only refer them to the provincial hospital so that they can be treated without harm”. (PVS_District02_Provider02)

Screening and detecting mental health care services were provided in the psychiatric unit in the provincial hospital or psychiatric at the national hospital. The confirmed case would be referred to the provincial psychiatric hospital for treatment.

Recently, mental health has been increasingly received attention due to highlighted cases with severe consequences (depression with deaths, particularly related to postnatal depression), bringing the Ministry of Health focused on mental health, including maternal mental health. In addition, mental health issues had been in concern since the coronavirus disease (COVID-19) appeared. The coronavirus disease could create a significant policy window to launch a public awareness campaign on mental health issues. That advocacy would drive policy changes at local and national levels that were intended to have long-term, trajectory-altering impacts on improving mental health services. During interviews, health policymakers and managers voiced support for integrating mental health services into existing maternal healthcare systems.

“In the past two years, mental health cases are increasing because of the COVID-19 pandemic. The pressure is stronger on women to take care of their families, deal with the economic crisis, social distancing, have a disease, and particularly bearing a baby. It is a big issue. We should consider it as a cause of maternal death. Yet, its consequences are magnified.” (Health policy maker 4)

Policymakers highlighted the need for accurate data on the burden of mental health issues at different levels, including national and local health facilities to advocate for the integration of the program. Evidence on the feasibility and effectiveness of the integration of mental health screening and counseling into the maternal health care system was also needed to develop policy proposals.

#### Post-partum care

Mothers were advised to visit hospitals for post-natal care. There was a protocol to check mothers who had pre-eclampsia, eclampsia, or diabetes. However, there was no follow-up system to care for women in the postpartum period.

### Service delivery by the level of care and type of health facilities

#### Limited maternal services utilization in district and commune health facilities in big cities

An interview with healthcare providers at the commune health center found that there were very few pregnant women who visit the commune health station for antenatal care, particularly in the cities where there were a lot of both public and private clinics and public hospitals on obstetrics. Unlike the commune health stations in rural areas, those belonged to the urban districts could not support delivery, women preferred higher health facilities for receiving comprehensive care:

“No one comes to check up here. It used to be, but since 2014, when the commune upgraded to the ward, the station couldn't support delivery anymore, so no one came to the antenatal care, no one for counseling.” (PVS_Commune02_Provider1)

“Currently, we want to strengthen the non-communicable disease management at commune health stations. But now the management of pregnant and postpartum mothers here is in crisis, frankly speaking, it is very bad. Only 10% to 20% of health stations have pregnant women visiting commune health stations for antenatal care. These are stations with skilled midwives or obstetrician-gynecologist technicians, more or less recognized by their community. People often do not go to the commune health station. They often choose a district hospital or a private clinic that is also a specialist in obstetrics, they are skilled, have a brand name or they even select a provincial or higher level of care”. (PVS_CDC02_Manager03)

District hospitals in big cities like Hanoi and HCMC also had very few women visited for pregnancy care:

“Even district-level hospitals have now receded. Now some district-level hospitals such as Tam Binh Hospital, such a large class II hospital, have an obstetric department but still have no patients. Go Vap Hospital's obstetrics department also started to recede, which means that no customers or patients are visiting for antenatal care, some just come for a regular gynecological examination” (PVS_District01_Manager01)

Some commune health stations and district health facilities organized screening for hypertension or diabetes under the NCD program, however, pregnant women were not a target group:

“Currently, there are a few communes that have programs [screening for hypertension or diabetes] they still do. For a long time, they have focused on non-communicable diseases for the elderly, but not for pregnant women.” (PVS_District02_Provider01)

Pregnant women who had diabetes or hypertension were also referred to a higher level of care for receiving treatment and management, not in the commune health station. As a result, commune health stations rarely provide personal counseling on non-communicable diseases or treatment or management for pregnant women.

“Q: How many pregnant women visit the commune health station for antenatal care?

A: Very few! They come here for tetanus vaccination only and may ask some questions. They have their private obstetrician. They had their phone number and must be counseled by them. They also look for information from the internet, there are a lot of communication channels nowadays.” (PVS_Commune 03_Provider01)

Some commune health stations collaborated with the Department of Reproductive Health in the District Health Center to screen cervical cancer for women aged 35 – 60 under the pilot program in some districts.

“We only screened for cervical cancer in previous years. The health station does not have a cervical cancer screening room, so it is often coordinated with the Reproductive Health Department of the District Health Center. For normal cases, women will be referred to the health station to be screened again next year, abnormal cases are moved up and reported to the health station for management, but those cases are not many.” (PVS_CDC01_Manager01)

The guidelines for breast cancer counseling and screening were just developed in 2021 (Decision 1639/QĐ-BYT dated 19.03.2021) and breast cancer screening has not yet been implemented at the primary health care level.

#### Preference for private sectors

The private sectors including private hospitals and clinics also make maternal health care services, particularly antenatal care more available and accessible. Pregnant women preferred private over public sectors for various advantages which included less waiting time and administrative requirements, better attitudes from healthcare providers, better equipment (advanced and updated ultrasound techniques, for example), and their ability to select healthcare providers.

“The trend now is that people will go to private clinics [for antenatal care] because the obstetrician has his clinic. It has the AT vaccine, and a test, and the obstetrician directly supports giving birth also. When women experience some labor signs, they call the obstetrician, and they are there instructed on how and when to be hospitalized. It has a good thing, it doesn't take time. If you have to wait, it takes very little time. The hospital is very crowded.” (PVS_Commune01_Provider01)

Integration of non-communicable of diseases into maternal health care services needs to take the private sector into account.

### Counseling and health promotion

As mentioned above, pregnant women would receive counseling for using pre-eclampsia screening packages and information about diabetes. In general, pregnant women would receive information on non-communicable diseases screening and prevention except for mental health issues:

“Diabetes is almost 100% consulting, the issue of whether they agree to use the screening service is up to their finance. 100% of women who come for antenatal care have their blood pressure measured. Nutrition counseling is also concerned, Breast cancer, cervical cancer, and breastfeeding are also advised. The mental health issues are still limited, from both the counselor and the people”. (PVS_POH01_Manager01)

Time for counseling was limited:

“Pregnant women are fully consulted. There is a protocol for the steps in antenatal check-ups that we must follow without saying anything. But this hospital is the last level of care, it is difficult to consult patients as limited time, so counseling is also limited.” (PVS_NOH02_Manager01)

“The number of patients is too large, so consultation and communication are limited. Medical staff don't have enough time for this.” (PVS_NOH03_Manager01)

However, some obstetricians said that they could decide the number of patients each day and time constrain were not a reason.

“Actually, it's the doctor who talks to the patient, in an easy-to-understand way, the patient will decide very quickly, mostly following the doctor’s advice. In our hospital, we don't have too much pressure in time, whether we want to do it fast or slow is the doctor's choice.” (PVS_NOH03_Provider01)

The implementation of counseling, therefore, was mainly based on each obstetrician. There was a monitoring system but worked ineffectively:

“There are also times when someone comes to supervise but usually does not have the professional capacity to supervise or the doctor glares and is afraid to leave.” (PVS_NOH01_Provider02)

At commune health stations, among the nine steps of the antenatal check-up procedure by the Ministry of Health, counseling was mentioned as the most skipped or weak step:

“In the case of having a large number of patients, the consultation stage is not thorough. If having fewer patients, they have more time to consult. If having more patients, they often do not carefully consult or do it with a whole group and then do the check-up.” (PVS_District02_Provider01)

Pregnant women who had a positive test with raised blood pressure or diabetes would receive more detailed and appropriate counseling than those who had not.

At national obstetric hospitals, health promotion and marketing for the service packages were focused to attract more patients. The information was available on their websites, youtube, Facebook, leaflets, and the billboards in the hospitals. Information on high blood pressure and diabetes was the most visible, followed by pre-marital services, breast and cervical cancer, and breastfeeding.

Commune Health Station provides health education and promotion about non-communicable diseases in general, breast and cervical screening and prevention, and breastfeeding through mass media such as loud-speaker, Facebook, webpage, and Zalo group but for the general population and does not focus on pregnant women (except breastfeeding). The provincial level and lower levels of care need more supports in creating and producing health education materials.

The health information on NCDs supported obstetricians a lot in proving their services but not enough. Health promotion needed to be strengthened to improve the literacy of pregnant women and their family members on non-communicable diseases:

“Regarding high blood pressure and diabetes, the difficulty is not about finance, it's about perception. Patients don't understand so people don't cooperate.” (PVS_POH01_Manager01)

“Patients do not trust our advice and do not follow our advice, especially about nutrition. For example, after eating, they are still hungry, so they eat more. The meals prepared by the hospital have been balanced and nutritious, but we buy extra food from outside. Then the patient's family members do not agree or support the treatment. They don’t trust the doctor. They agree with what you said but do not follow your advice.” (PVS_NOH01_Provider02)

## Finance

### Limited budget and mechanism for allocating budget was a barrier in implementing screening NCDs program including for women at the commune and district level

In the 2021 – 2025 period, the state budget for implementing both the NCD program and reproductive health program was limitedly allocated. A health manager at the center level explained the reason for cutting down:

“Funding resources for operation management and maintenance of the information and reporting system must be regulated. In the long term, it must be from the state budget, not by programs and projects. Programs and projects have a short-term period, after achieving the goal of the project, the investor withdraws, the activities will also end.”(PVS_CDC02_Manager03)

The implementation of those programs mainly relied on local government. Each commune, district, and provincial health facility would make an implementation plan and request for a budget from their commune/district/provincial People's Committee, respectively.

“In 2022, the Department of Health directed the allocation of funds to districts. From this year [2023], the district health facilities are required to budget and apply for having a budget. So from this year, HCDC only budgeted for HCDC activities.” (PVS_CDC02_Manager01)

In big cities including Hanoi and Hochiminh city, budgets were available for implementation however NCDs were in priorities for funding but not reproductive or maternal health care services.

“Budget is available. But because Hanoi has already reached the targets of sustainable development, the indicators of maternal mortality, under-five mortality, and under-one-year-old mortality have all reached the target. Hanoi's indicators are similar to developed countries. MOH recommends antenatal check-ups at least 4 times, surely Hanoi must be 10 times.” (PVS_CDC03_Manager01)

In Hochiminh CDC for example, maternal health care was funded only on updated training on obstetrics, nutrition, cervical screening, treatment of mother-child interaction, evaluating hospital procedures, and monitoring breastfeeding.

Even though NCDs were being priorities for funding in some provincial people’s committees, the screening was implemented with a very limited number of participants:

“In 2018, we coordinated with NGOs to organize the screening in the community, but after that the project ended and we didn't have any more funding. The cost of screening is very large. Nowadays, we usually set a small target, each commune health station will screen for 100-200 women a year, for example” (PVS_CDC01_Manager01)

The limited budget was also mentioned as a barrier to implementing the community screening program:

“Now the programs have no funding, before they get 20-30K (~$1 – 1.5) to come, if we have some medicine for them, they joint, if not, they don't. When people see they receive nothing, their attitudes change negatively a bit. The targets are now all elderly, they need to have something to come, some kind of gift. Old and young are alike actually. Many times when we implement community programs, we have to pay out of pocket for people. Sometimes it makes it difficult to work.” (PVS_Commune01_Provider01)

Before 2020, cervical cancer screening and counseling were provided for free under the national project. After 2020, co-payment, if any was at an acceptable level. Screening for cervical cancer may contribute to promoting cervical cancer prevention at the primary health care level.

“The screening sometime was free of charge. I was responsible for inviting women. If it is free, they will come. But now that the state and people share, it can be a co-payment. Compared to the hospital, the price is not high and they do not have to wait and receive enthusiastic advice. However, if I have a target of 100 women, I have to invite more than half that number to exclude those who don't come.”(PVS_Commune 03_Provider01)

It should be noted that not all local governments have the budget for health as in big cities like Hanoi and HCMC.

“For some localities that depend on the state budget, the Ministry of Health must be responsible for them to have national target programs to support poverty and inequity reduction, particularly in mountainous ethnic minorities provinces.” (PVS_MOH02)

The delay in funding allocated was a barrier to organized cervical screening in the community:

“In 2022, the funding was allocated very late, so we could not implement prevention activities in the commune health station, we could only organize some training activities at HCDC to keep it on schedule.” (PVS_CDC01_Manager01)

“This year, HCDC only budgeted for HCDC but the funding process was slow, so it hasn't implemented anything yet” (PVS_CDC02_Manager01)

### Hospital autonomy and the growing up of private clinics/hospitals

The growth of private clinics and hospitals and the fiscal autonomy of public hospitals on the one hand made maternal services available and the competition among health facilities improved the quality of services, particularly in counseling.

“In general, the hospital in the previous years was quite crowded, now many private clinics are opened, the number of patients is reduced. They prefer to visit private clinics. Fewer patients come to hospitals and doctors have more time to give advice.”(PVS_POH01_Provider 02).

As mentioned above, the number of patients assigned to obstetricians each day was under the control of the obstetrician, and it was not affected by their performance evaluation or income. Some obstetricians manifested that good counseling was not only bringing benefits for the hospitals but only for their own private after official working hours.

“I spend time on counseling because if the patient agrees to use the screening packages, it increases the benefit to the hospital. Besides, it also helps to increase the number of patients for my private practice” (PVS_NOH01_Provider01)

On the other hand, as maternal health services were not covered by health insurance, maternal health services in general and NCD counseling and screening services were available but only accessible to those who could pay for them:

“In fact, public hospitals have a lot of customers. There will be customers with good economic conditions and those with poor ones. Counseling those service packages needs to be considered by customers’ economic status. For example, at week 12 of pregnancy, according to the procedure, we can provide prenatal screening like a Double test or NIPT, thyroid screening, or pre-eclampsia screening. The total cost of those screenings is also about 4-5 million. It very much depends on the financial status of the customer, not everyone can pay for it.” (PVS_NOH03_Provider01)

To increase the coverage of counseling, screening, and treatment of NCDs within maternal health care and reduce inequality in accessibility, there was a need for developing a list of services covered by health insurance:

“As for screening and consulting activities, the most difficult thing now is that it is not paid by health insurance.” (PVS_MOH01)

“We do not have a list of services ourselves, we need to issue service packages for prevention, screening, and early detection of non-communicable diseases, which are paid for by the state budget besides health insurance. I think it is necessary to make such a list, and how much it costs, so on that basis, the quotas should be assigned to the commune health stations for them to implement and evaluate.” (PVS_MOH02)

## Drugs and medical supplies

Disruption of drug and medical supplies had been reported in health facilities at the commune and district levels and provincial general hospitals:

“However, drugs are uneven supplying. The drug supply is irregular and not timely, for example, it is expected at the beginning of the year, but it will only be available from June to July, even at the end of the year. Thus, patient management is not maintained. This year there are 1000 hypertensive patients managed for example, but maybe next year there will be only 500. People come here once, or twice but receive no medicine, they would go to a higher level.” (PVS_CDC02_Manager01)

Disruption of drug and medical supplies at lower levels of care also contributed to the preference for having antenatal visits at higher levels one. Drugs and medical supplies were available at national and provincial obstetrics hospitals without disruption.

“For drugs, the pharmacy and bidding department is still able to handle it, not to the case of no medicine for treating patients… It is a specialized hospital and the number of drugs needed is small, so we still can manage it.” (PVS_POH01_Provider02)
